# Neurofibromatosis Symptom-Lacking B-Cell Lineage Acute Lymphoblastic Leukemia with Only an *NF1* Gene Pathogenic Variant

**DOI:** 10.3390/diagnostics13081486

**Published:** 2023-04-20

**Authors:** Zehwan Kim, Jong Ho Lee

**Affiliations:** Department of Laboratory Medicine, College of Medicine, Yeungnam University, Daegu 42415, Republic of Korea

**Keywords:** acute lymphoblastic leukemia, ALL, B-ALL, BCP-ALL, Ph-like ALL, BCR-ABL-like ALL, *NF1*, neurofibromatosis, Ras pathway

## Abstract

Next-generation sequencing technology has improved molecular genetic analysis, and many molecular genetic studies have been utilized for diagnostic classification, risk stratification, and prognosis prediction of acute lymphoblastic leukemia (ALL). Inactivation of neurofibromin or Nf1, a protein derived from the *NF1* gene, causes Ras pathway regulation failure, which is related to leukemogenesis. Pathogenic variants of the *NF1* gene in B-cell lineage ALL are uncommon, and in this study, we reported a pathogenic variant that is not registered in any public database. The patient diagnosed with B-cell lineage ALL had no clinical symptoms of neurofibromatosis. Studies on the biology, diagnosis, and treatment of this uncommon disease, as well as other related hematologic neoplasms, such as acute myeloid leukemia and juvenile myelomonocytic leukemia, were reviewed. Biological studies included epidemiological differences among age intervals and pathways for leukemia, such as the Ras pathway. Diagnostic studies included cytogenetic, FISH, and molecular tests for leukemia-related genes and ALL classification, such as Ph-like ALL or BCR-ABL1-like ALL. Treatment studies included pathway inhibitors and chimeric antigen cell receptor T-cells. Resistance mechanisms related to leukemia drugs were also investigated. We believe that these literature reviews will enhance medical care for the uncommon diagnosis of B-cell lineage ALL.

Acute lymphoblastic leukemia (ALL) is a neoplasm of lymphoid blood cells presenting with uncontrolled proliferation of immature lymphoid cells predominantly in children and adults. Although almost 80% of ALL pediatric and adult patients are treated, it is still a major cause of mortality and morbidity [1].

The World Health Organization (WHO) published a revision of the “WHO Classification of Tumors of Hematopoietic and Lymphoid Tissues” in 2008 and another in 2016 [2]. The revisions aimed to additionally reflect the results of molecular genetic studies, along with clinical presentation, microscopic presentation of the blood, immunophenotypic studies, and cytogenetic studies. In particular, because of next-generation sequencing (NGS), molecular genetic analysis has significantly improved, and many molecular genetic studies have been utilized for diagnostic classification, risk stratification, and prognosis prediction [2].

According to the WHO and ALL researchers, most ALL is classified as B-cell lineage ALL and less frequently as T-cell lineage ALL. Both ALL lineages are further classified into different types of genetic abnormalities, including chromosomal structure aberrations and pathogenic genetic sequencing variants [3]. The relationship between genetic abnormalities and ALL has been extensively studied in order to elucidate the mechanism of ALL development, risk stratification, treatment selection, and prognosis prediction [4].

Neurofibromin (Nf1), the protein produced by the *NF1* gene, modulates the activity of the Ras pathway, which delivers signals from the granulocyte-monocyte colony-stimulating factor to proliferating cells. The *NF1* gene-derived protein suppresses the conversion of the activated complex, Ras-GTP, which negatively regulates the RAS/MAPK pathway to the inactivated complex, Ras-GDP [5]. Inactivation of the *NF1* gene due to somatic (acquired) pathogenic sequence variants results in leukemogenesis [6]. Pathogenic variants in the *NF1* gene causing Nf1 inactivation have been reported in pediatric acute myeloid leukemia (AML) and T-cell lineage ALL [6], and are associated with poor prognosis [7].

Inactivation of Nf1 derived from the *NF1* gene pathogenic variant causing Ras pathway regulation failure in B-cell lineage ALL is uncommon [3,4], and it is necessary to confirm *NF1* gene inactivation in B-cell lineage ALL for risk stratification and prognosis prediction. Hence, in this study, we reported an *NF1* gene pathogenic variant in B-cell lineage ALL in patients with no clinical neurofibromatosis symptoms.

A 30-year-old female patient, with no neurofibromatosis symptoms, visited the emergency department with fever and nausea. She had a temperature of 37 °C, icteric sclera, RUQ tenderness, major depressive disorder, and corresponding medication. A complete blood cell study showed a white blood cell count 840/uL, Hb of 9.7 g/dL, and platelet count 79,000/uL. PT-INR was 1.36 and C-reactive protein was 1.011 mg/dL. A chemistry study showed total protein 5.47 g/dL, albumin 3.35 g/dL, total bilirubin 8.2 mg/dL, aspartate aminotransferase 1933 IU/L, alanine aminotransferase 2519 IU/L, alkaline phosphatase 165 IU/L, and lactate dehydrogenase 2834 IU/L. She had splenomegaly, identified by CT study. A peripheral blood smear study showed 59% lymphocytes and 20% blasts. A bone marrow (BM) smear study identified 98% blasts (Figure 1), and an immunophenotyping study identified 99.36% CD10, 99.97% CD19, 94.08% CD20, 98.57% CD22, 25.44% cytoplasmic CD22, 0.06% CD23, and 88.25% cCD79a, all of which complied with the B-cell lineage. The karyotype study identified 46,XX,add(16)(q12) [7].

To identify genetic abnormalities, we performed RT-PCR (HemaVision, DNA Diagnostic, Risskov, Denmark), and the results were negative for major and minor *BCR*/*ABL1*, *ETV6*/*RUNX1*, and *TCF3*/*PBX1*. In an NGS study using the Illumina MiSeq Dx system (Illumina, San Diego, CA, USA) with a panel of 47 genes, *IKZF1*, *JAK2*, *NRAS*, *RB1*, *TP53*, *ABL1*, *BRAF*, *BTG1*, *CDKN2A*, *CREBBP*, *CRLF2*, *DNM2*, *DNMT3A*, *ETV6*, *EZH2*, *FBXW7*, *FLT3*, *GATA3*, *IDH1*, *IDH2*, *IL7R*, *JAK1*, *JAK3*, *KDM6A*, *KMT2A*, *KMT2D*, *KRAS*, *LEF1*, *LMO1*, *MAPK1*, *NF1*, *NOTCH1*, *NT5C2*, *PAX5*, *PHF6*, *PTEN*, *PTPN11*, *RUNX1*, *SETD2*, *SH2B3*, *STAG2*, *STAT3*, *STAT5B*, *TBL1XR1*, *TCF3*, *TPMT*, and *WHSC1* (*NSD2*), the results showed only one variant. The variant was an *NF1* gene sequence indel variant, NM_001042492.2:c.4691_ 4698delinsGGCCCTCCC, with 80% variant allele frequency (VAF) (Figure 2), which is a frameshift variant and is clinically interpreted as Tier 2 (potential clinical significance) [8]. The corresponding indel variant was confirmed via Sanger sequencing (Figure 3). A variant with VAF above 50% can be a homozygous one, if the NGS platform suffers from technological limitations such as guanine–cytosine content (GC–content) proportion of the target sequence or PCR bias, and especially if the NGS platform is an amplicon-based sequencer. We performed Sanger sequencing of the target sequence with the variant. We concluded that the variant was heterozygous with VAF 80%. After the examination, the patient was transferred to another hospital without treatment.

Molecular studies on ALL diagnosis play an important role in risk classification, targeted therapy approaches, and prognosis prediction. Germline gene abnormalities in the *NF1* gene are known to cause autosomal dominant neurofibromatosis [9]. However, in our case report, the patient had no neurofibromatosis symptoms due to germline *NF1* gene variants. Although no attempt was made to identify the germline variant in the patient, NGS showed that the *NF1* gene has only one variant, identified as VAF at 80%; thus, it is assumed to be a somatic variant of the *NF1* gene. The *NF1* gene variant NM_001042492.2(NF1):c.4691_4698delinsGGCCCTCCC was discovered in the patient with B-cell lineage ALL without clinical neurofibromatosis symptoms, and this indel variant was reported neither in COSMIC [10] nor ClinVar [11]. As the found *NF1* gene variant is a frame-shift mutation, which causes a truncated Nf1 protein, it is a likely oncogenic variant and classified into Tier 2 variants [8]. Yeung et al. described the genetic abnormalities in B-cell lineage ALL by classifying them into three categories using diagnostic methods. First, genetic abnormalities were identified using cytogenetic testing; second, those not found through cytogenetic testing but in the FISH test; and third, genetic abnormalities found through molecular diagnosis [12]. In aneuploid ALL, mostly identified using karyotyping, specific gene variants are identified as specific aneuploidy types. In a review of 124 hypodiploid pediatric ALL cases, 70.6% of near-haploid ALLs (24–31 chromosomes) were associated with the receptor tyrosine kinase pathway or Ras pathway, which includes *NRAS*, *KRAS*, *NF1*, *PTPN11*, and *FLT3* genes [12,13]. Furthermore, *NF1* gene abnormality is related to myocyte enhancer factor 2D (*MED2D*) gene rearrangement, as well as *TP53* gene abnormalities [12]. In this patient, the *TP53* gene was wildtype, but the *NF1* gene was malfunctioning. Additionally, poor prognosis due to *NF1* gene variants inactivating Nf1 has often been reported in AML and T-cell lineage ALL [5,6,14]. Inherited activity deficiency of mismatch repair genes and B-cell lineage ALL results in a high tumor mutation burden (TMB), and after conventional chemotherapy, TMB can increase rapidly by up to two times [15]. In this patient’s NGS examination, the TMB was not calculated using whole-exome or whole-genome sequencing. However, since only one small mutation was observed in the *NF1* gene out of the 47 ALL genes tested, it can be assumed that there was no high TMB. In the ALL study, the age range of young adults was up to 29 years of age in the European Union and up to 40 years of age in the USA; hyperdiploid B-cell lineage ALL has been observed in 30–40% of patients with pediatric ALL but in less than 5% of young adults [16]. This patient was 30 years old, 46,XX,add(16)(q12) [7] was observed in the karyotype test, and both major and minor *BCR*/*ABL1* were confirmed as negative. Abnormalities were identified in the *NF1* gene, suggesting a modification in the Ras pathway, resulting in Ph-like ALL of the B-cell lineage [17]. Ph-like ALL, which accounts for approximately 15% of ALL cases regardless of cell lineage, has a similar gene expression pattern to Philadelphia chromosome-positive ALL, Ph-positive ALL, or *BCR*-*ABL1* ALL, and has a poor prognosis [18,19]. Ph-like ALL is characterized by sustained activation of kinase or cytokine receptor signaling, and Ofran et al. suggested the term kinase-derived ALL or KD-ALL instead of Ph-like ALL [18]. Ph-like ALL is divided into two major frequency subgroups and other minor frequency subgroups. The first major subgroup is an *ABL*-class fusion containing genes such as *ABL1*, *ABL2*, *CSF1R*, and *PDGFRB*, similar to *BCR*-*ABL1*. The second major subgroup contains *CRLF2*, *JAK2*, and *EPOR* genes, which continuously activate JAK/STAT signaling. One of the last minor subgroups includes the Ras pathway, including abnormalities in *BLNK*, *DGKH*, *FGFR1*, *IL2RB*, *LYN*, *NTRK3*, *PDGFRA*, *PTK2B*, *TYK2*, and *NF1* genes [19]. In 328 patients with de novo AML, when exons 2, 3, 4, and 11 of *PTPN11*, exons 8 and 9 of *CBL*, exons 1 and 2 of *NRAS*, and all *NF1* exons were sequenced, *NF1* gene abnormalities leading to changes in the Ras pathway were found in 2.1% of de novo patients [20] and poor prognosis was observed. *NF1* gene abnormality was found together with a complex karyotype in de novo AML and this coincidence makes it difficult to determine whether the abnormality of this gene initiates leukemia or whether it is caused by disease progression [21]. However, in this patient, only the *NF1* gene was abnormal among the 47 genes, which can help to determine the onset or progression of disease. Blast cells expressed 99.97% CD19 in the immunophenotyping test of this patient, which supports the use of tisagenlecleucel, a chimeric antigen receptor T cell [22]. Since the Ras pathway is abnormal due to a loss of function of the *NF1* gene, farnesyltransferase inhibitor, or MEK pathway (which follows the Ras pathway) inhibitors such as trametinib, selumetinib, or cobimetinib may be considered [19,23,24,25]. Treatment approaches related to other hematologic neoplasms associated with the Ras pathway should also be considered. In juvenile myelomonocytic leukemia, a rare and aggressive type of pediatric leukemia, Ras pathway hyperactivation has been reported with a high frequency of approximately 80–90%, and many studies are being conducted on classification, treatment protocols, and clinical trials. These treatment approaches can be used as a reference when treating B-cell lineage ALL in which the Ras pathway has abnormalities [20,25,26]. Since the patient was transferred to another hospital immediately after diagnosis, it was not possible to confirm which treatment protocol was used for this ALL case and whether resistance developed. However, because NGS was performed during diagnosis, we identified the loss of function of only one gene, *NF1*, among the 47 genes tested. If relapse and drug resistance were observed after complete remission, any abnormality of the same or different gene, or the same or different pathway, could be investigated [27]. Although diagnostic tests such as BM smear, immunophenotype, and karyotype studies, as well as ALL gene panel NGS sequencing were performed, the chances of recurrence are unknown because the patient was transferred to another hospital. Additionally, although the ALL gene panel NGS test was performed on 47 genes, this is not sufficient to judge whether there was only *NF1* gene abnormality as neither whole-exome nor whole-genome sequencing was performed. A further limitation is that copy number variations or structural abnormalities were not identified.

In conclusion, we sequenced 47 genes in a B-cell lineage ALL case and identified an uncommon and Tier 2 somatic variant in the *NF1* gene only, and the variant was not registered with COSMIC or ClinVar. We believe that the literature reviews related to this case will enhance medical care for this uncommon diagnosis of B-cell lineage ALL.

## Figures and Tables

**Figure 1:**
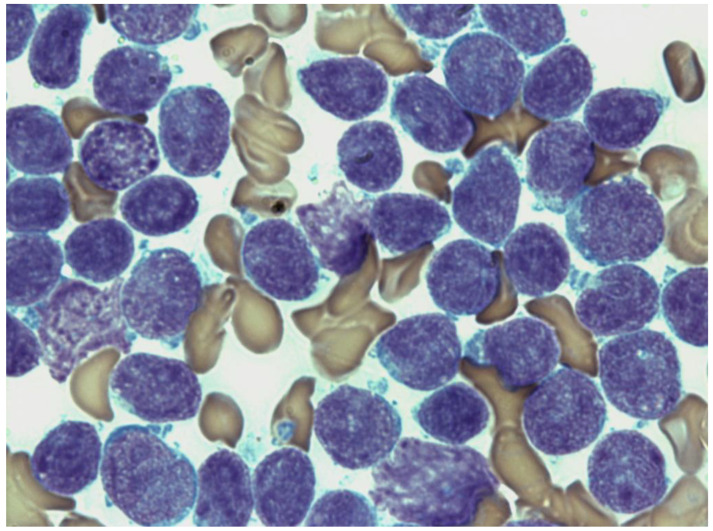
Bone marrow hematological cells from aspirate smear sample. The number of small- to medium-sized blasts with scant cytoplasm increased in up to 98% of absolute nucleated cells and the number of megakaryocytes and their precursors decreased. The granulocytic series and the erythroid series cells were also suppressed. (Wright stain, ×1000).

**Figure 2:**
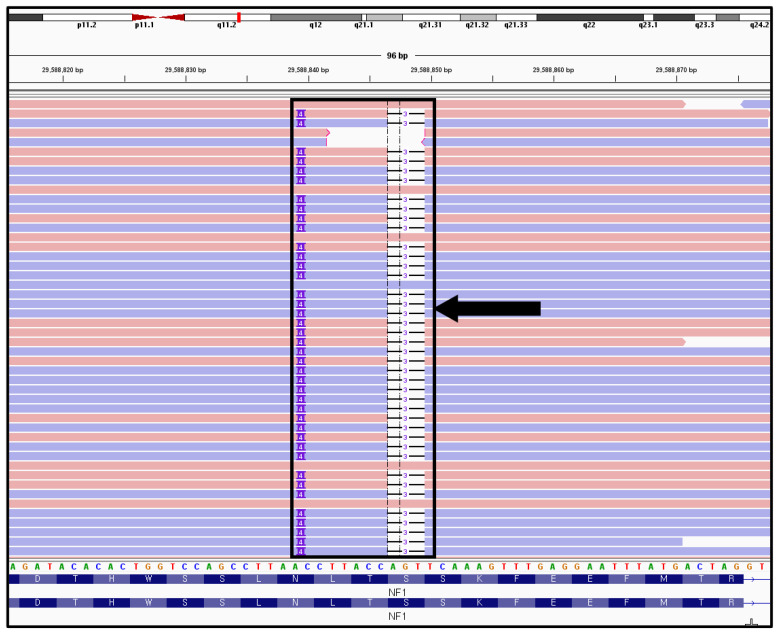
Screen capture of the Integrative Genomic Viewer tool. It shows the content of the Binary Alignment Map (BAM) file, obtained from NGS study, from the position 29,588,800 to 29,588,895 (hg19 version reference genome) of chromosome 17. The block arrow and box indicates the indel variant NM_001042492.2(NF1):c.4691_4698delinsGGCCCTCCC. The BAM file was generated by Illumina MiSeq Dx system, which uses hybridization with oligonucleotide probes for target enrichment.

**Figure 3:**
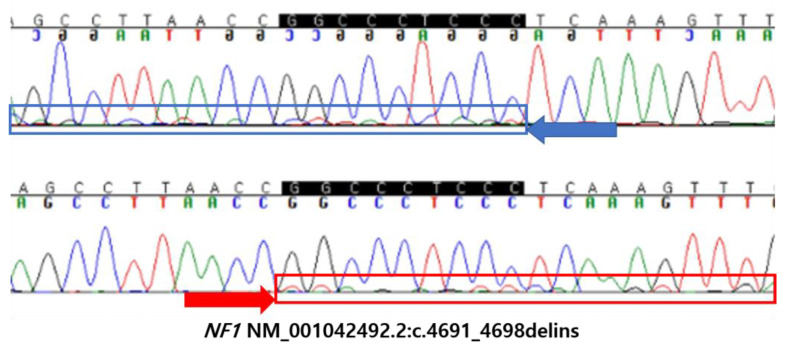
Corresponding indel variant via Sanger sequencing. The same indel variant as in Figure 2 can be confirmed by its forward sequence with the indel variant (red arrow and box) and the reverse sequence with the indel variant (blue arrow and box). We conclude that the variant is somatic because of the following four reasons. Initially, we could not find any neurofibromatosis symptom including café-au-lait spots, freckling, or multiple cutaneous neurofibromas. However, pathogenic germline *NF1* mutation has almost 100% penetrance to neurofibromatosis [9]. Therefore, we thought the patient did not have the variant before the onset of the leukemia. Secondly, neurofibromatosis is an autosomal dominant inherited disorder [9]. This fact also lowers the possibility of germline mutation. Thirdly, variant allele frequency (VAF) of the found *NF1* gene variant was 80%, which is not around 50% for heterozygous nor around 100% for homozygous germline variants. Finally, Sanger sequencing shows overlapped sequences which are the mix of wild-type sequence and the indel variant-induced sequence (in the red as well as blue boxes). If the variant is germline heterozygous, the overlapped sequence (in the red and blue boxes) should show that some nucleotides have almost the same amplitude of the two difference sequences. If the variant is germline homozygous, the overlapped sequence (in the red and blue boxes) should show only one indel variant-induced sequence. The found *NF1* variant c.4691_4698delins(p.Leu1564Argfs*7) was an indel one, which consists of 8 base-pair deletion followed by 9 base-pair insertion. It causes frame-shift during translation from mRNA to protein and it introduces stop-codon after 7 codons. The result is the formation of a truncated Nf1 protein.

## Data Availability

Not applicable.
